# The association between triglyceride glucose-body mass index and all-cause mortality in critically ill patients with atrial fibrillation: a retrospective study from MIMIC-IV database

**DOI:** 10.1186/s12933-024-02153-x

**Published:** 2024-02-10

**Authors:** Yi Hu, Yiting Zhao, Jing Zhang, Chaomin Li

**Affiliations:** 1 Department of Cardiology, Honghui Hospital of Xi’an Jiaotong University, 555 Youyi East Road, Xi’an, Shaanxi, PR China; 2 Department of Cardiology, The Second Affiliated Hospital of Air Force Medical University, Xi’an, PR China

**Keywords:** Atrial fibrillation, Triglyceride glucose-body mass index (TyG-BMI), Insulin resistance, Prognosis, All-cause mortality

## Abstract

**Background:**

The TyG-BMI index, which is a reliable indicator of insulin resistance (IR), has been found to have a significant correlation with the occurrence of cardiovascular events. However, there still lacks study on the TyG-BMI index and prognosis in patients with atrial fibrillation (AF). The objective of the present study was to evaluate the relationship between TyG-BMI index at admission to ICU and all-cause mortality in critically ill patients with AF.

**Methods:**

The patient’s data were extracted from Medical Information Mart for Intensive Care IV(MIMIC-IV) database. All patients were divided into four groups according to TyG-BMI index. Outcomes include primary and secondary endpoints, with the primary endpoint being the 30-day and 365-day all-cause mortality and the secondary endpoint being the 90-day and 180-day all-cause mortality. TyG-BMI index was quartile and Kaplan-Meier curve was used to compare the outcome of each group. Cox proportional-hazards regression model and restricted cubic splines (RCS) were conducted to assess the relationship between TyG-BMI index and outcomes.

**Results:**

Out of a total of 2509 participants, the average age was 73.26 ± 11.87 years, with 1555 (62.0%) being males. Patients with lower level of TyG-BMI had higher risk of 30-day, 90-day, 180-day and 365-day all-cause mortality, according to the Kaplan-Meier curves (log-rank *P* < 0.001). In addition, cox proportional-hazards regression analysis revealed that the risk of 30-day, 90-day, 180-day and 365-day all-cause mortality was significantly higher in the lowest quartile of TyG-BMI. Meanwhile, the RCS analysis indicated that L-typed relationships between TyG-BMI index and all-cause mortality, with inflection points at 223.60 for 30-day and 255.02 for 365-day all-cause mortality, respectively. Compared to patients with TyG-BMI levels below the inflection points, those with higher levels had a 1.8% lower risk for 30-day all-cause mortality (hazard ratio [HR] 0.982, 95% confidence interval [CI] 0.9676–0.988) and 1.1% lower risk for 365-day all-cause mortality (HR 0.989, 95% CI 0.986–0.991).

**Conclusion:**

In critically ill patients with AF, a lower TyG-BMI level is significantly associated with a higher risk of 30-day, 90-day, 180-day and 365-day all-cause mortality. TyG-BMI index could be used as a valid indicator for grading and treating patients with AF in the ICU.

**Supplementary Information:**

The online version contains supplementary material available at 10.1186/s12933-024-02153-x.

## Introduction

Over the past few decades, cardiovascular disease has ranked as the world’s leading cause of death. In 2021, 20.5 million people died from cardiovascular disease (CVD), accounting for about one-third of all deaths worldwide [1]. As a common CVD, atrial fibrillation (AF) is associated with a significantly increased risk of all-cause mortality, heart failure (HF), hospitalization, and thromboembolic events [2, 3]. The conditions of critically ill patients admitted to intensive care units (ICUs) are complex and a variety of causes may be involved. A study revealed that AF occurred in around 14% of all patients who were admitted into ICU [4]. However, there are lacking studies which demonstrate the prognosis of critically ill patients with AF. Therefore, it is vital to identify and avoid risk factors to reduce complications and mortality in critically ill patients with AF.

Insulin resistance (IR) has been linked to AF in previous study [5]. As a side effect of IR, the body secretes excessive insulin as compensation for a reduction in insulin’s efficiency to increase glucose uptake and utilization. This results in hyperinsulinemia, which maintains blood glucose levels. IR is the typical feature of metabolic syndrome and type 2 diabetes [6]. The current gold standard for evaluating IR is the hyperinsulinemic euglycemic clamp (HEC), which is applicable to varieties type of populations [7]. Thus, the homeostasis model assessment-estimated insulin resistance (HOMA-IR) index is the most accurate, but the technique requires special equipment and skilled technicians, is expensive and time-consuming, requires multiple blood sampling during the test, is difficult to be accepted by patients, is currently only used for scientific research, and cannot be applied on a large scale in the clinic, and the method of this test is to determine the body’s sensitivity to exogenous insulin, which has the problem of bioefficacy. Nowadays, the triglyceride-glucose (TyG) index was proposed to be a simple surrogate marker of IR, and it has been proved to predict the prognosis of cardiovascular disease, including coronary heart disease (CHD), HF, acute myocardial infarction, stroke and hypertension [8–10]. More importantly, recent studies have shown that the TyG index, when combined with obesity indicators such as body mass index (BMI), waist-to-height ratio (WtHR), and waist circumference (WC), could significantly improve the validity of assessing IR [11]. There have been reported that a combination of TyG and BMI could perform better in accordance with HOMA-IR for IR assessment [12], and demonstrated that TyG-BMI index may be closely associated with the prevalence of hypertension, cardiovascular outcomes in patients with coronary artery disease, and non-alcoholic fatty liver disease [13–15]. However, there still lacks studies on the influence of the TyG-BMI index on the outcomes of patients with AF, especially those who were critically ill patients with AF.

Therefore, the purpose of the current study was to assess the potential impact of the baseline TyG-BMI index at admission to ICU on all-cause mortality in critically ill patients with AF. The finding of this study may help to explore new methods for early identification and improvement of prognosis in critically ill patients with AF.

## Methods

### Source of data

The study is a retrospective analysis, which data were extracted from the large publicly available critical care database-Medical Information Mart for Intensive Care IV (MIMIC-IV, version 2.2). The MIMIC-IV database, with some improvements over MIMIC-III, including data updates and some table reconstruction, collects clinical data from more than 190,000 patients admitted and 450,000 hospitalizations recorded from 2008 to 2019 at the Beth Israel Deaconess Medical Center (BIDMC, Boston, MA, United States). The database records detailed information on patient’s demographics, laboratory tests, medications, vital signs, surgical operations, disease diagnosis, medication management, and follow-up survival status. In order to obtain data access, we have studied training course of National Institutes of Health (NIH) for protecting human study participants and passed the tests of the Collaborative Institutional Training Initiative. A waiver of informed consent was granted because the database did not contain protected information and the patients were anonymous.

### Study design and population

**Fig. 1:**
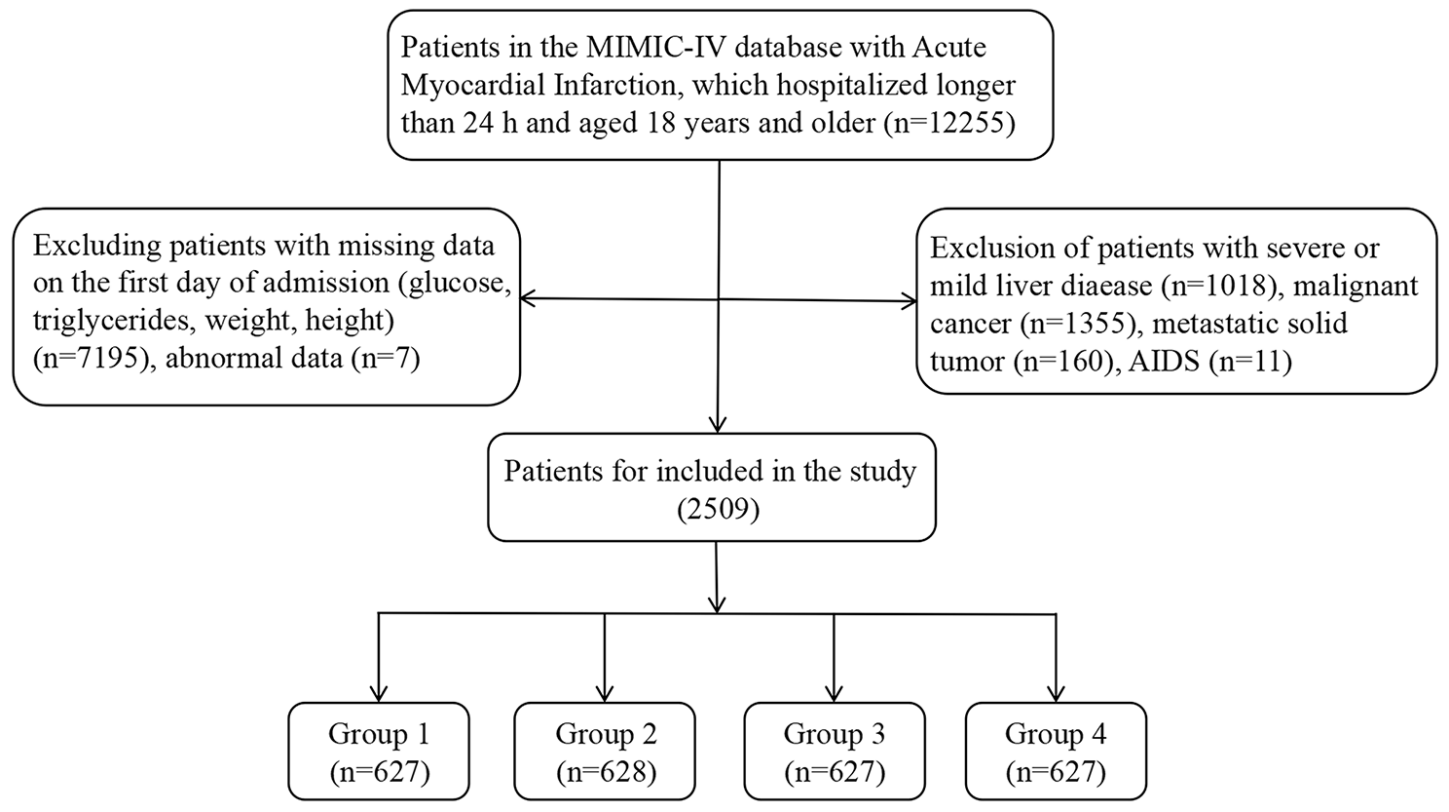
Flowchart of the selection of patients

Patients with AF who were hospitalized and admitted into ICU for the first time were included in the study. A total of 12,255 patients with AF were categorized diagnoses using codes from both the International Classification of Diseases, Ninth Revision (ICD-9) and Tenth Revision (ICD-10). The ICD 9 and ICD 10 code of AF in the study including 42,731, I48, I480, I481, I482, I489, I4811, I4819, I4820, I4821, I4891. The exclusion criteria were as follows: (1) patients stayed in ICU less than 24 h; (2) multiple admissions to the ICU for AF, for whom only data from the first admission were extracted; (3) insufficient data (such as serum fasting blood glucose, triglycerides, weight, height and abnormal data); (4) patients with severe or mild liver diseases, malignant cancer, metastatic solid tumor and acquired immune deficiency syndrome (AIDS). A total of 2509 patients were included in the final study cohort and divided into four groups according to the quartiles of the TyG-BMI index (Fig. 1).

### Data extraction

Navicat Premium (Version 16.1.15) was used to extract data using structure query language (SQL). A large amount of data about each patient at admission were extracted, including demographic information (age, gender, race/ethnicity, height, weight, body mass index [BMI], smoking); past history (acute myocardial infarction [AMI], hypertension, diabetes, chronic obstructive pulmonary disease [COPD], congestive heart failure [CHF], peripheral vascular disease [PVD], cerebrovascular disease [CVD], dementia, rheumatic disease); vital sign (systolic blood pressure [SBP], diastolic blood pressure [DBP], mean blood pressure [MBP], heart rate [HR], respiratory rate [RR]); medication (aspirin, clopidogrel, beta blockers, angiotensin-converting enzyme inhibitors/angiotensin receptor blockers [ACEI/ARB], calcium channel blocker [CCB], digitalis, diuretics, amiodarone, insulin, statin, dabigatran, rivaroxaban, heparin, warfarin); laboratory data (SpO_2_, PO_2_, PaCO_2_, pH, base excess [BE], anion gap, bicarbonate, glucose, serum urea [BUN], creatinine, calcium, chloride, sodium, potassium, prothrombin time [PT], partial prothrombin time [PTT], hematocrit, hemoglobin, platelets, white blood cell [WBC], lymphocytes, neutrophils, mean corpuscular hemoglobin [MCH], mean corpusular hemoglobin concentration [MCHC], mean corpuscular volume [MCV], red blood cell [RBC], red cell distribution width [RDW], HbA1c, high density lipoprotein [HDL], low density lipoprotein[LDL], total cholesterol [TC], triglyceride [TG], glasgow coma scale [GCS], simplified acute physiology score [SAPSII], sequential organ failure assessment [SOFA]); event (cardiac arrest, cardiogenic shock); length of stay (LOS) (LOS in hospital, LOS in ICU); outcome (30-day mortality, 90-day mortality, 180-day mortality, 365-day mortality). All blood indicators were measured for the first time after the patients were admitted into ICU. Variables with more than 20% missing values were excluded. Variables with missing values less than 20% were filled with missing values using multiple interpolation.

### Outcomes

The outcome of patients with AF in MIMIC-IV database including 30-day mortality, 90-day mortality, 180-day mortality, 365-day mortality. The primary outcomes of present study were 30-day and 365-day all-cause mortality. Secondary outcomes were 90-day and 180-day all-cause mortality.

### Calculation of TyG-BMI

The TyG index was calculated as ln[fasting glucose (mg/dl)×fasting TG (mg/dl)]/2 [9]. BMI was calculated as body weight (Kg)/height^2^ (m). The TyG-BMI index was determined based on the combination of TyG index and BMI. TyG-BMI index was computed according the equation: TyG index×BMI [12].

### Statistical analysis

Testing for normality of continuous variables was conducted first, and Student t-tests and one-way ANOVAs were used to identify data that conformed to a normal distribution, then the data were expressed as mean ± standard deviation (SD). Non-normally distributed data were tested using the Wilcoxon rank-sum test and expressed in median with interquartile range (IQR). Chi-square or Fisher exact tests were used to analyze categorical variables expressed in absolute numbers with percentages.

By stratifying by TyG-BMI index, Kaplan-Meier (K-M) curves were used to determine the incidence rate of major and secondary outcomes. Univariable Cox analysis were performed to assess the relationships between TyG-BMI index and 30-day, 90-day, 180-day and 365-day mortality. The multivariate Cox proportional-hazard regression model included variables that were clinically relevant or which had a univariate relationship with outcome. The variables included in the final model were carefully chosen based on the number of events available. Model 1 only included the TyG-BMI index, while model 2 adjusted for age, gender, race, heart rate, hypertension, diabetes, insulin, beta blockers, statin, amiodarone, digitalis, pH, PT, platelets, chloride, potassium, RBC, WBC, BUN, creatinine, SOFA. In both models, the lowest quartile of the TyG-BMI index was used as the reference. The TyG-BMI index was also analyzed as a continuous variable using restricted cubic splines (RCS) to clarify the dose-effect correlations with the risk of major and secondary outcome events. If the correlations were non-linear, then the recursive algorithm was used to calculate the inflection points between the TyG-BMI index and 30-day, 90-day, 180-day and 365-day mortality. To further examine the relationship between TyG-BMI index and 30-day, 90-day, 180-day and 365-day mortality, the two-segment Cox proportional risk model was applied on both sides of the inflection point. Additionally, stratified analysis was performed based on gender, age (< 60 years old or ≥ 60 years old), race/ethnicity, hypertension and diabetes.

All statistical analysis were processed using SPSS software (version 22.0, IBM Corporation, United States) and R software (version 4.3.2, R Foundation for Statistical Computing, Austria), *P* < 0.05 was considered statistically significant.

## Results

### Baseline characteristics of study individuals

In the study, 12,255 patients with AF were included in MIMIC-IV database, and total of 2509 patients with AF met the inclusion criteria and were analyzed. The patients enrolled in the study had a mean age of 73.26 ± 11.87 years and the percentage of male was 62.0%. Based on the quartiles of the TyG-BMI index at admission (Q 1: < 233.94, Q 2: 233.94–274.70, Q 3: 274.70-325.91, Q 4: >325.91), the study participants’ baseline characteristics were analyzed and presented in Table 1. The levels of TyG-BMI index of the four groups were 209.38 (193.71 to 222.77), 255.10 (244.86 to 263.84), 297.11 (285.16 to 310.74) and 373.57 (345.04 to 418.95), respectively. Participants with lowest TyG-BMI index were older, lower prevalence of AMI, diabetes, hypertension, higher prevalence of COPD, CVD, dementia, lower usage rate of beta blockers, diuretics, insulin, statin, heparin, higher usage rate of aspirin, lower levels of HR, PaCO_2_, anion gap, glucose, creatinine, potassium, hemoglobin, WBC, lymphocytes, neutrophils, RBC, HbA1c, TG, TC, LOS in hospital, LOS in ICU, and higher levels of SpO_2_, PO_2_, pH, chloride, PTT, MCH compared to the higher group. Meanwhile, the 30-day mortality (18.5% vs. 12.3% vs. 12.4% vs. 13.1%, *P* < 0.01), 90-day mortality (25.7% vs. 17.5% vs. 16.3% vs. 17.1%, *P* < 0.01), 180-day mortality (30.5% vs. 22.0% vs. 20.9% vs. 19.3%, *P* < 0.01) and 365-day mortality (36.2% vs. 25.2% vs. 24.4% vs. 23.0%, *P* < 0.01) in Q1 group were all higher than other three groups, and there were no differences among Q 2, Q 3 and Q 4 groups.

**Table 1:** Baseline characteristics of patients grouped according to TyG-BMI index quartiles

Variables	Q 1 < 233.94 (*N* = 627)	Q 2 233.94–274.70 (*N* = 628)	Q 3 274.70-325.91 (*N* = 627)	Q 4 > 325.91 (*N* = 627)	P
**Demographics**					
Age, years	77.36 ± 12.20	75.55 ± 10.74	72.82 ± 10.74	67.30 ± 11.26	< 0.01
Male, n (%)	353 (56.3)	401 (63.9)	421 (67.1)	380 (60.6)	< 0.01
Weight, Kg	64.05 ± 11.19	77.24 ± 11.50	89.42 ± 12.85	113.83 ± 25.42	< 0.01
Race, White	448 (71.5%)	480 (76.4%)	472 (75.3%)	438 (69.9%)	0.08
Height, cm	168.95 ± 10.41	169.36 ± 10.92	170.52 ± 10.48	169.92 ± 11.15	0.06
BMI, Kg/m2	22.33 ± 2.50	26.81 ± 1.88	30.63 ± 2.34	39.43 ± 8.19	< 0.01
Smoking, n (%)	39 (6.2)	33 (5.3)	32 (5.1)	33 (5.3)	0.81
**Past history, n (%)**					
AMI	450 (71.8%)	507 (80.7%)	513 (81.8%)	504 (80.4%)	< 0.01
Hypertension	450 (71.8%)	507 (80.7%)	513 (81.8%)	504 (80.4%)	< 0.01
Diabetes	125 (19.9%)	171 (27.2%)	253 (40.4%)	320 (51%)	< 0.01
COPD	209 (33.3%)	163 (26%)	177 (28.2%)	198 (31.6%)	0.02
CHF	308 (49.1%)	288 (45.9%)	316 (50.4%)	323 (51.5%)	0.21
PVD	123 (19.6%)	112 (17.8%)	122 (19.5%)	101 (16.1%)	0.33
CVD	162 (25.8%)	146 (23.2%)	141 (22.5%)	117 (18.7%)	0.02
Dementia	32 (5.1%)	23 (3.7%)	23 (3.7%)	11 (1.8%)	0.02
Rheumatic disease	25 (4%)	16 (2.5%)	31 (4.9%)	26 (4.1%)	0.17
**Vital signs**					
SBP, mmHg	121.59 ± 25.68	122.11 ± 24.31	119.46 ± 22.70	120.61 ± 25.13	0.24
DBP, mmHg	64.82 ± 16.87	65.47 ± 18.28	64.98 ± 17.64	66.70 ± 18.66	0.24
MBP, mmHg	81.78 ± 17.13	82.29 ± 18.32	80.93 ± 18.91	82.50 ± 18.91	0.43
HR, beats/min	85.45 ± 19.91	85.47 ± 20.24	87.87 ± 20.04	88.44 ± 21.01	0.01
RR, times/min	18.22 ± 5.70	18.08 ± 5.96	18.43 ± 5.58	18.67 ± 5.72	0.29
**Medication, n (%)**					
Aspirin	475 (75.8%)	500 (79.6%)	512 (81.7%)	459 (73.2%)	< 0.01
Clopidogrel	107 (17.1%)	101 (16.1%)	93 (14.8%)	78 (12.4%)	0.12
Beta blockers	538 (85.8%)	561 (89.3%)	567 (90.4%)	540 (86.1%)	0.02
ACEI/ARB	55 (8.8%)	54 (8.6%)	54 (8.6%)	40 (6.4%)	0.35
CCB	67 (10.7%)	63 (10%)	70 (11.2%)	74 (11.8%)	0.78
Digitalis	96 (15.3%)	76 (12.1%)	84 (13.4%)	79 (12.6%)	0.36
Diuretics	481 (76.7%)	531 (84.6%)	534 (85.2%)	539 (86%)	< 0.01
Amiodarone	286 (45.6%)	297 (47.3%)	309 (49.3%)	298 (47.5%)	0.64
Insulin	374 (59.6%)	437 (69.6%)	451 (71.9%)	477 (76.1%)	< 0.01
Statin	391 (62.4%)	425 (67.7%)	456 (72.7%)	404 (64.4%)	< 0.01
Dabigatran	9 (1.4%)	9 (1.4%)	13 (2.1%)	13 (2.1%)	0.69
Rivaroxaban	20 (3.2%)	16 (2.5%)	22 (3.5%)	23 (3.7%)	0.69
Heparin	521 (83.1%)	518 (82.5%)	545 (86.9%)	555 (88.5%)	< 0.01
Warfarin	276 (44%)	294 (46.8%)	320 (51%)	294 (46.9%)	0.10
L**aboratory data**					
SpO_2_, %	99.00 (96.00 to 100.00)	99.00 (96.00 to 100.00)	99.00 (95.00 to 100.00)	98.00 (95.00 to 100.00)	< 0.01
PO_2_, mmHg	223.50 (72.00 to 399.00)	259.00 (81.50 to 395.00)	195.00 (74.00 to 370.00)	131.00 (65.00 to 312.00)	< 0.01
PaCO_2_, mmHg	40.00 (36.00 to 45.00)	40.00 (36.00 to 45.00)	41.00 (37.00 to 47.00)	44.00 (39.00 to 51.00)	< 0.01
pH	7.40 (7.34 to 7.44)	7.40 (7.35 to 7.44)	7.38 (7.33 to 7.42)	7.36 (7.30 to 7.41)	< 0.01
BE, mmol/L	0.00 (-2.00 to 2.00)	0.00 (-2.00 to 2.00)	0.00 (-2.00 to 1.00)	0.00 (-3.00 to 2.00)	0.04
Anion gap, mmol/L	14.43 ± 4.61	14.33 ± 4.47	14.77 ± 4.76	15.33 ± 4.90	< 0.01
Bicarbonate, mmol/L	23.49 ± 4.49	23.36 ± 4.46	22.72 ± 4.36	23.49 ± 5.05	0.01
Glucose, g/dL	116.00 (99.00 to 142.00)	125.00 (105.00 to 151.00)	129.00 (109.00 to 169.00)	143.00 (117.00 to 195.50)	< 0.01
BUN, mg/dL	21.00 (15.00 to 32.00)	21.00 (15.00 to 33.00)	22.00 (16.00 to 36.00)	23.00 (16.00 to 38.50)	< 0.01
Calcium, mg/dL	8.48 ± 0.78	8.48 ± 0.84	8.43 ± 0.85	8.43 ± 0.96	0.63
Chloride, mEq/L	104.31 ± 6.86	104.33 ± 6.73	104.36 ± 6.34	102.84 ± 6.87	< 0.01
Creatinine, mg/dL	1.00 (0.70 to 1.40)	1.00 (0.80 to 1.50)	1.10 (0.90 to 1.60)	1.20 (0.90 to 1.80)	< 0.01
Sodium, mEq/L	138.57 ± 5.24	138.45 ± 4.68	138.39 ± 4.60	138.23 ± 5.12	0.69
Potassium, mEq/L	4.32 ± 0.80	4.29 ± 0.80	4.40 ± 0.82	4.44 ± 0.90	< 0.01
PT, s	15.20 (12.90 to 18.40)	15.10 (13.10 to 18.30)	14.80 (12.90 to 18.30)	14.95 (13.20 to 17.50)	0.71
PTT, s	32.25 (28.20 to 39.40)	32.30 (28.00 to 40.30)	31.70 (27.50 to 41.70)	30.80 (27.30 to 37.10)	0.02
Hematocrit, %	32.39 ± 6.98	32.58 ± 7.10	33.39 ± 7.33	33.78 ± 7.37	< 0.01
Hemoglobin, g/dL	10.60 ± 2.30	10.75 ± 2.39	10.95 ± 2.52	10.99 ± 2.44	0.01
Platelets, K/µL	211.96 ± 113.94	198.29 ± 103.58	201.86 ± 90.88	206.65 ± 88.60	0.08
WBC, K/µL	11.74 ± 7.21	11.75 ± 5.67	12.41 ± 5.65	13.24 ± 6.90	< 0.01
Lymphocytes, K/µL	1.35 ± 0.92	1.51 ± 0.92	1.65 ± 1.24	1.62 ± 1.25	< 0.01
Neutrophils, K/µL	10.03 ± 7.30	9.66 ± 5.08	10.46 ± 5.55	11.11 ± 6.24	< 0.01
MCH, pg	30.09 ± 2.47	30.27 ± 2.45	29.82 ± 2.51	29.55 ± 2.45	< 0.01
MCHC, %	32.77 ± 1.66	33.06 ± 1.62	32.83 ± 1.61	32.55 ± 1.75	< 0.01
MCV, fL	91.88 ± 6.53	91.62 ± 6.42	90.87 ± 6.49	90.87 ± 6.84	< 0.01
RBC, K/µL	3.54 ± 0.78	3.57 ± 0.79	3.69 ± 0.84	3.74 ± 0.83	< 0.01
RDW, %	14.83 ± 2.01	14.66 ± 1.91	14.82 ± 2.14	14.88 ± 1.91	0.21
HbA1c	5.97 ± 0.93	6.16 ± 1.28	6.44 ± 1.30	6.89 ± 1.79	< 0.01
HDL, mg/dL	51.00 (41.00 to 63.00)	47.00 (38.00 to 59.00)	43.00 (35.00 to 56.00)	41.00 (33.00 to 52.00)	< 0.01
LDL, mg/dL	81.00 (59.00 to 104.00)	79.00 (57.00 to 107.00)	82.00 (60.00 to 103.00)	84.00 (61.00 to 112.00)	0.47
TG, mg/dL	82.00 (63.00 to 115.00)	104.00 (77.00 to 142.00)	122.00 (88.00 to 173.00)	155.00 (106.00 to 227.50)	< 0.01
TC, mg/dL	155.00 (124.00 to 184.00)	151.00 (124.00 to 189.00)	153.00 (126.00 to 186.00)	159.00 (131.50 to 195.50)	0.01
TyG-BMI	209.38 (193.71 to 222.77)	255.10 (244.86 to 263.84)	297.11 (285.16 to 310.74)	373.57 (345.04 to 418.95)	< 0.01
GCS	14.00 (10.00 to 15.00)	14.00 (10.00 to 15.00)	14.00 (10.00 to 15.00)	14.00 (9.00 to 15.00)	0.80
SAPSII	39.00 (32.00 to 46.00)	38.00 (31.00 to 47.00)	39.00 (31.00 to 49.00)	39.00 (31.00 to 49.00)	0.54
SOFA	5.00 (3.00 to 7.00)	5.00 (3.00 to 8.00)	6.00 (4.00 to 9.00)	6.00 (4.00 to 10.00)	< 0.01
**Event**, n (%)					
Cardiac arrest	21 (3.3%)	26 (4.1%)	29 (4.6%)	33 (5.3%)	0.40
Cardiogenic shock	55 (8.8%)	50 (8%)	70 (11.2%)	58 (9.3%)	0.25
**Length of stay (LOS)**					
LOS in hospital	9.16 (5.99 to 14.77)	9.12 (6.07 to 15.73)	10.27 (6.43 to 15.94)	10.87 (6.63 to 17.55)	< 0.01
LOS in ICU	3.04 (1.77 to 6.05)	3.40 (1.82 to 6.69)	3.58 (2.03 to 7.52)	4.13 (2.02 to 8.82)	< 0.01
**Outcome**					
30-day mortality, n (%)	116 (18.5%)	77 (12.3%)	78 (12.4%)	82 (13.1%)	< 0.01
90-day mortality, n (%)	161 (25.7%)	110 (17.5%)	102 (16.3%)	107 (17.1%)	< 0.01
180-day mortality, n (%)	191 (30.5%)	138 (22.0%)	131 (20.9%)	121 (19.3%)	< 0.01
365-day mortality, n (%)	227 (36.2%)	158 (25.2%)	153 (24.4%)	144 (23.0%)	< 0.01

Continuous variables are presented as mean ± SD if normally distributed, and median (interquartile range) if not normally distributed. Categorical variables are presented as number of patients (%). BMI, body mass index; AMI, acute myocardial infarction; COPD, chronic obstructive pulmonary disease; CHF, congestive heart failure; PVD, peripheral vascular disease; CVD, cerebrovascular disease; SBP, systolic blood pressure; DBP, diastolic blood pressure; MBP, mean blood pressure; HR, heart rate; RR, respiratory rate; PaCO_2_, carbon dioxide partial pressure; PaO_2_, partial pressure of arterial oxygen; BE, base excess; BUN, urea nitrogen; PT, prothrombin time; PTT, partial prothrombin time; WBC, white blood cell; MCH, mean corpuscular hemoglobin; MCHC, mean corpusular hemoglobin concerntration; MCV, mean corpuscular volume; RBC, red blood cell; RDW, red cell distribution width; HDL, high density lipoprotein; LDL, low density lipoprotein; TC, total cholesterol; TG, triglyceride; GCS, Glasgow coma scale; SOFA, sequential organ failure assessment; SAPS II, simplified acute physiology score

### Study outcomes

**Fig. 2:**
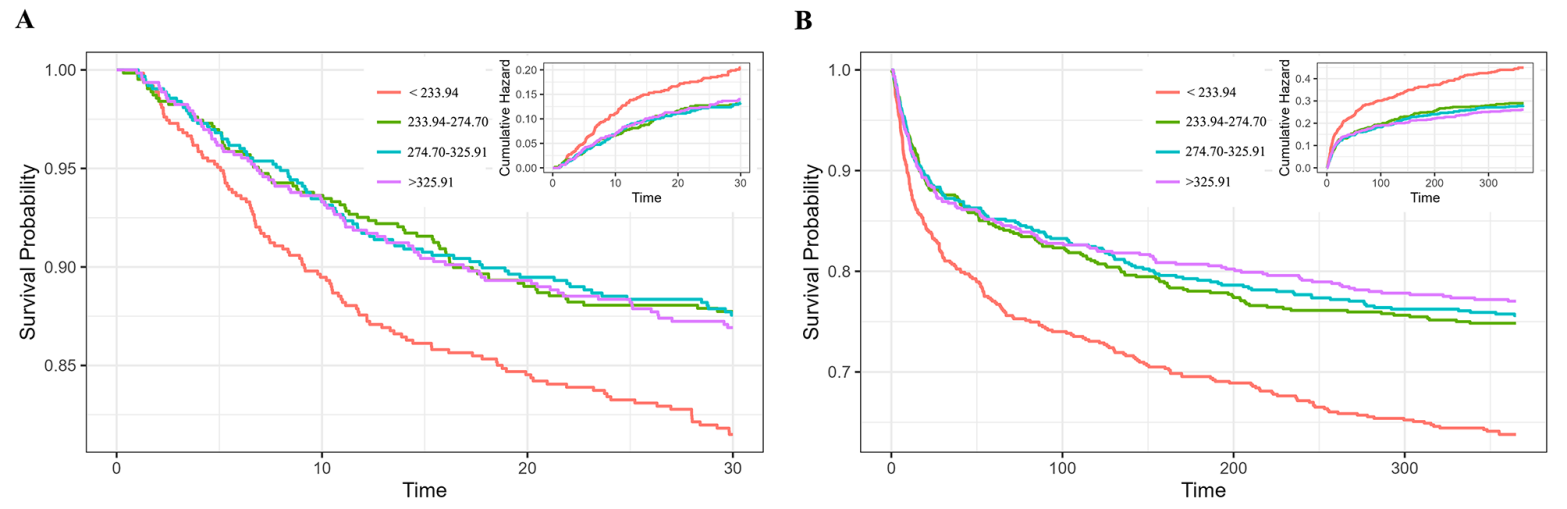
Kaplan–Meier survival analysis curves for all-cause mortality Kaplan–Meier curves and cumulative incidence of 30-day (**A**) and 365-day (**B**) all-cause mortality stratified by TyG-BMI index

The K-M curve indicated that the different prevalence of 30-day mortality and 365-day mortality among the four groups by TyG-BMI quartiles (Fig. 2), as well as the differences in 90-day mortality and 180-day mortality among the four groups (Supplementary Fig. 1). Patients with lowest TyG-BMI index demonstrated the lowest 30-day, 90-day, 180-day and 365-day survival rate compared to those with higher levels of TyG-BMI index (log-rank *P* < 0.001). However, there was no disparity in 30-day, 90-day, 180-day and 365-day survival rate among the other three groups.

### Relationship between TyG-BMI and clinical outcomes of patients with AF

In order to investigate the independent effects of TyG-BMI index on mortality, two Cox regression models were applied (Table 2). After adjustment for age, gender, race, heart rate, hypertension, diabetes, insulin, beta blockers, statin, amiodarone, digitalis, pH, PT, platelets, chloride, potassium, RBC, WBC, BUN, creatinine, SOFA (Model 2), the adjusted hazard ratios (HR) and 95% confidence intervals (CIs) from lowest to highest TyG-BMI index categories (< 233.94, 233.94–274.70, 274.70-325.91, > 325.91) were 1.00 (reference), 0.67 (0.48–0.92), 0.68 (0.49–0.95), 0.65 (0.46–0.93), respectively, for 30-day all-cause mortality; 1.00 (reference), 0.70 (0.56–0.89), 0.67 (0.52–0.85), 0.57 (0.44–0.74), respectively, for 365-day all-cause mortality. We found that AF patients with lower levels of TyG-BMI index (< 233.94) had higher risk 30-day and 365-day all-cause mortality than those with TyG-BMI index ≥ 233.94. Meanwhile, as shown in Supplementary Table 1, we also detected the relationship between TyG-BMI index and 90-day and 180-day all-cause mortality, and the analogous results were obtained.

**Table 2:** Cox proportional hazard models for 30-day and 365-day all-cause mortality

	TyG-BMI index			
	Q 1	Q 2	Q 3	Q 4
**30-day mortality**				
Number of deaths (%)	116 (18.50)	77 (12.28)	78 (12.44)	82 (13.08)
Model 1 HR (95% CI) P-value	1.00	0.64 (0.48–0.85) < 0.01	0.65 (0.49–0.86) < 0.01	0.68 (0.52–0.91) < 0.01
Model 2 HR (95% CI) P-value	1.00	0.67 (0.48–0.92) 0.01	0.68 (0.49–0.95) 0.02	0.65 (0.46–0.93) 0.02
**365-day mortality**				
Number of deaths (%)	227 (36.20)	158 (25.20)	153 (24.40)	144 (22.97)
Model 1 HR (95% CI) P-value	1.00	0.65 (0.53–0.79) < 0.01	0.62 (0.51–0.77) < 0.01	0.59 (0.48–0.72) < 0.01
Model 2 HR (95% CI) P-value	1.00	0.70 (0.56–0.89) ＜0.01	0.67 (0.52–0.85) < 0.01	0.57 (0.44–0.74)＜0.01

Model 1 Univariate model

Model 2 adjusted for Age, Gender, Race, Heart rate, Hypertension, Diabetes, Insulin, Beta blockers, Statin, Amiodarone, Digitalis, pH, PT, Platelets, Chloride, Potassium, RBC, WBC, BUN, Creatinine, SOFA.

### The detection of nonlinear relationships

**Fig. 3:**
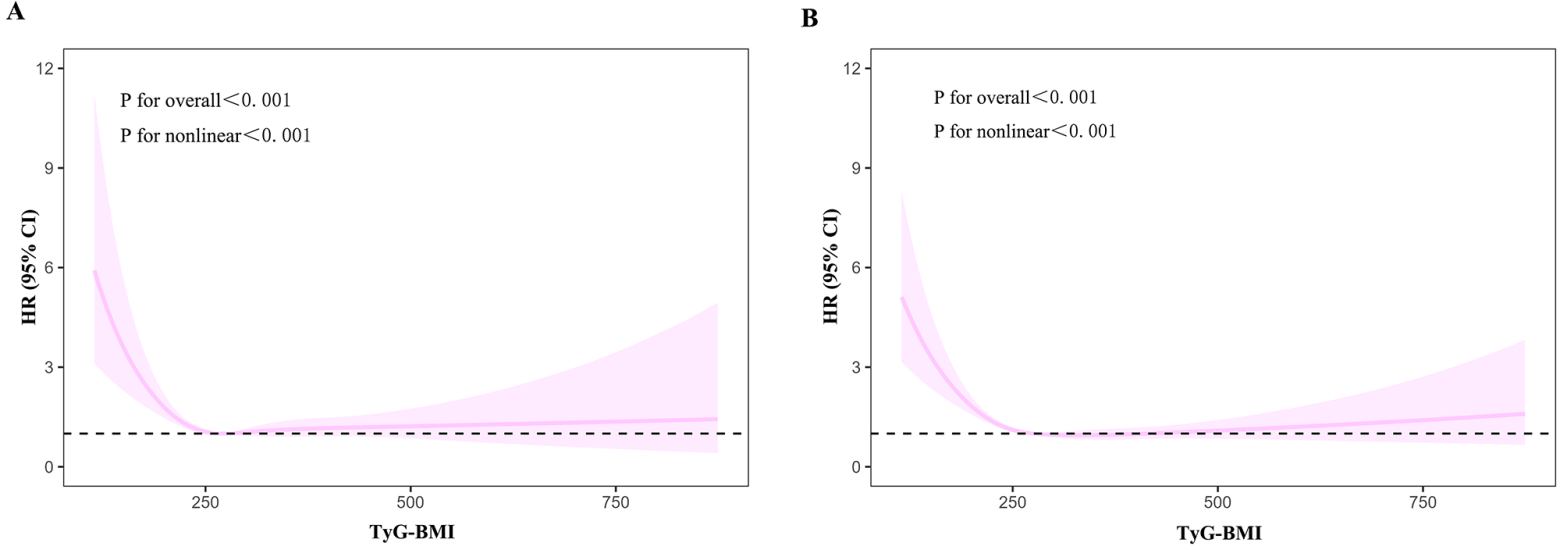
Restricted cubic spline regression analysis of TyG-BMI index with all-cause mortality Restricted cubic spline regression analysis of TyG-BMI index with 30-day (**A**) and 365-day (**B**) all-cause mortality

RCS curves were performed to detect the nonlinear relationship between TyG-BMI index and 30-day, 90-day, 180-day and 365-day all-cause mortality. We found that the L-typed associations between TyG-BMI index and 30-day and 365-day all-cause mortality (Fig. 3A, B), as well as 90-day and 180-day all-cause mortality (Supplementary Fig. 2A, B). Then the Cox proportional hazards models and a two-piecewise Cox proportional hazards model were combined to investigate the non-linear relation between TyG-BMI index levels and all-cause mortality in patients with AF (both *P* for log-likelihood ratio < 0.05) (Table 3, Supplementary Table 2). The inflection points for 30-day, 90-day ,180-day and 365-day all-cause mortality were identified as 223.60, 258.23, 229.85, 255.02, respectively. When TyG-BMI index were less than 223.60 or 255.02, a reduction of 1-unit in the TyG-BMI level correlated with a 1.8% and 1.1% increase in the risk of 30-day and 365-day all-cause mortality, respectively (HR 0.982; 95% CI 0.976, 0.988 and HR 0.989; 95% CI 0.986, 0.991, respectively). If the TyG-BMI index was below 258.23 or 229.85, a reduction of 1-unit in the TyG-BMI level corresponded to a 1.1% and 1.5% increase in the risk of 90-day and 180-day all-cause mortality, respectively (HR 0.989; 95% CI 0.986, 0.993 and HR 0.985; 95% CI 0.981, 0.990, respectively).

**Table 3:** Threshold effect analysis of TyG-BMI index on 30-day and 365-day all-cause mortality in AF patients

	HR (95% CI), P-value
**30-day mortality**	
Fitting by the standard linear model	0.998 (0.997−1.000) 0.02
Fitting by the two-piecewise linear model	
Inflection point	223.60
TyG-BMI < 223.60	0.982 (0.976–0.988) < 0.01
TyG-BMI ≥ 223.60	1.000 (0.999–1.002) 0.72
P for Log-likelihood ratio	< 0.01
**365-day mortality**	
Fitting by the standard linear model	0.998 (0.997–0.999) < 0.01
Fitting by the two-piecewise linear model	
Inflection point	255.02
TyG-BMI < 255.02	0.989 (0.986–0.991) < 0.01
TyG-BMI ≥ 255.02	1.000 (0.999–1.002) 0.59
P for Log-likelihood ratio	< 0.01

TyG-BMI, triglyceride glucose-body mass index; The inflection of threshold effect analysis of TyG-BMI index on 30-day all-cause mortality was 223.60; The inflection of threshold effect analysis of TyG-BMI index on 365-day all-cause mortality was 255.02

### Stratified analyses

**Fig. 4:**
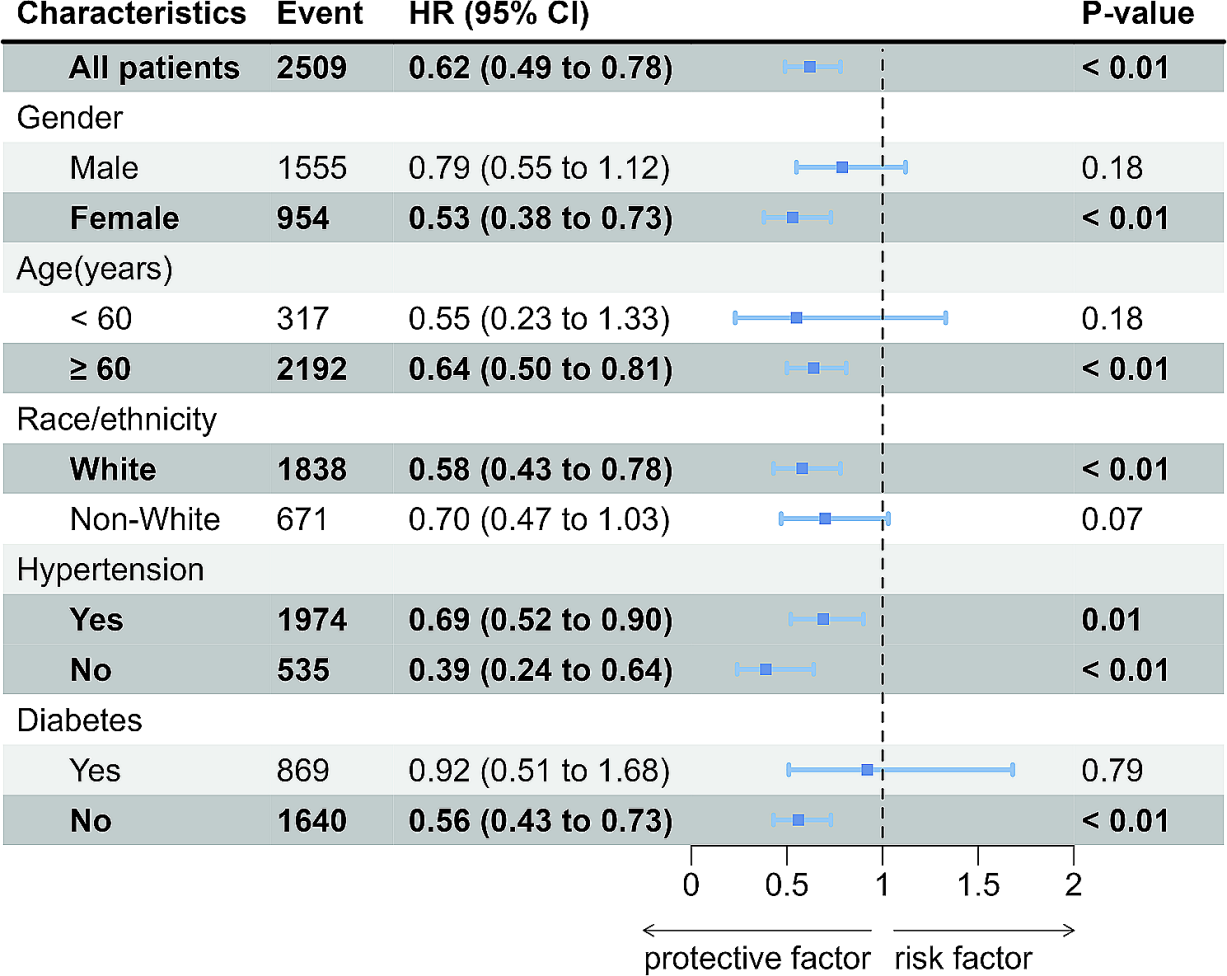
Forest plots of stratified analyses of TyG-BMI index and 30-day all-cause mortality

**Fig. 5:**
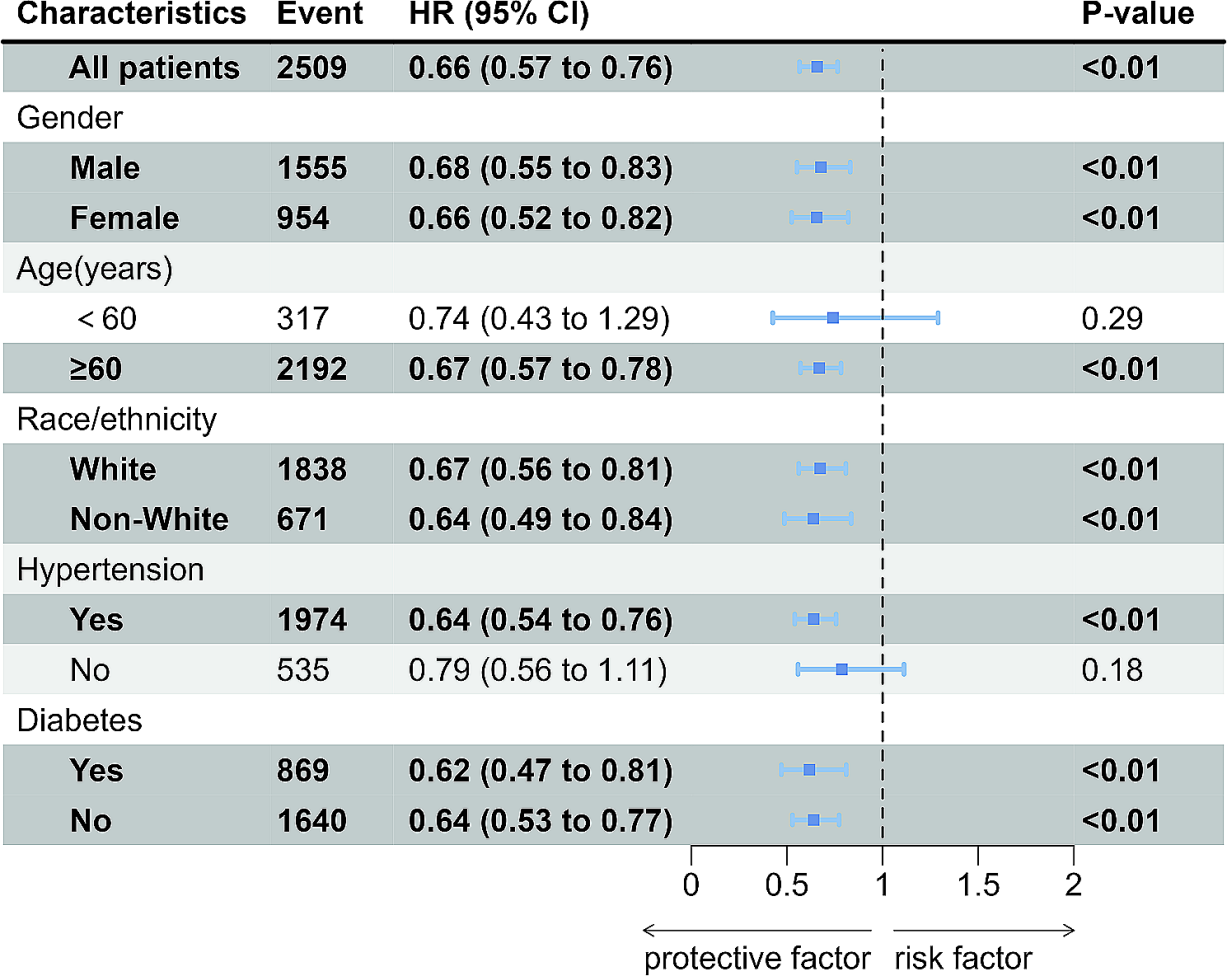
Forest plots of stratified analyses of TyG-BMI index and 365-day all-cause mortality

To further explore whether the relationships between TyG-BMI and 30-day, 90-day, 180-day and 365-day all-cause mortality persisted in different conditions, subgroups analysis were conducted for gender, age, race/ethnicity, hypertension and diabetes. The HRs of 30-day, 90-day, 180-day and 365-day all-cause mortality were all significant in subgroups of age ≥ 60 years and female, while there was no statistical significance in subgroups of age < 60 years and male (Figs. 4 and 5, Supplementary Fig. 3, Supplementary Fig. 4). The correlation between TyG-BMI levels and 90-day, 180-day and 365-day all-cause mortality were all statistically significant (*P* < 0.05) in patients with or without hypertension and diabetes, as well as the White or the non-White patients (Fig. 5, Supplementary Fig. 3, Supplementary Fig. 4). Whereas, the correlation between TyG-BMI levels and 30-day all-cause mortality were only statistically significant (*P* < 0.05) in patients without diabetes or the White patients (Fig. 4).

## Discussion

The present study was the first retrospective investigation to explore the association between TyG-BMI levels and all-cause mortality in critically ill patients with AF. We discovered the L-shaped relationships between TyG-BMI index levels and 30-day, 90-day, 180-day and 365-day all-cause mortality in the study, indicating that there was a significant association between lower TyG-BMI index levels and higher risk of mortality among critically ill patients with AF. These results may facilitate the development of clinical guidelines for reducing death among these patients.

IR is the typical characteristic of metabolic syndrome and obesity. As obesity rates increase globally, the incidence and prevalence of IR and related CVD are also accelerating [16]. It is reported that IR, which is a key risk factor for the development and progression of AF, can lead to remodeling of atrial structure and function, and abnormal intracellular calcium homeostasis, ultimately leading to the development and progression of AF [5]. Meanwhile, retrospective studies were conducted on patients who had previously undergone AF ablation by Wang et al. and found that IR was closely related to AF recurrence, and the more severe the status of IR, the higher the risk of AF recurrence [17]. Mechanisms of IR to AF include: increased vulnerability to induced AF, increased oxidative stress, increased interstitial fibrosis, impaired calcium homeostasis, inappropriate neurohumoral activation [5, 18, 19]. However, a study from Framingham Heart Study demonstrated that no significant association was observed between IR and AF events in a community-based cohort followed for 10 years [20]. We thought the reason was that the mean age of the study population was 59 years and the prevalence of cardiovascular risk factors was low. IR may need to be synergistic with other clinical factors of the metabolic syndrome to increase susceptibility to AF, such as hypertension, fasting glucose ≥ 100 mg /dL, IR, large waist circumference, low HDL, and high triglycerides. Noteworthy, the pathophysiology and underlying mechanisms of IR cause AF still not entirely determined.

The main feature of AF is very rapid and uncoordinated atrial activity. AF is classified as paroxysmal if it self-terminates within 7 days, persistent if it lasts continuously for > 7 days, long-standing persistent if it is present continuously for > 1 year, or as permanent (chronic) arrhythmia [21]. There are many mechanisms and risk factors for the occurrence of AF, including IR, smoking, hypertension, diabetes. Multiple risk factors influence cardiac afterdepolarizations, extra-systolic activity and atrial re-entry [22]. AF become increasingly persistent and resistant to therapy over time, the key mechanism by which this phenomenon occurs is atrial fibrosis. Previous studies indicated that systemic inflammation and oxidative stress caused by IR could induce atrial enlargement, inflammatory activation, local myocardial fibrosis, and electrical conduction abnormalities, all of which led to AF and promoted its persistence [23]. Therefore, there is no doubt that IR is a risk factor for the development and progression of AF.

TyG index, as a validated marker for assessing IR, had been reported to be strongly associated with AF and to be an independent risk factor for the onset and development of AF in hospitalized patients [24]. Liu et al. found a U-shaped association between TyG index and the incidence of AF in the general population by studying a cohort after 24 years of follow-up [25]. Numerous studies have demonstrated the usefulness and effectiveness of the TyG-BMI index for assessing IR [26, 27]. Recently, studies found that TyG-BMI could be a simple and solid index to assess the risk of major adverse cardiovascular events (MACEs), and higher TyG-BMI index was related to an increased incidence of MACEs [15, 28]. In addition, Chen L et al. also found that TyG-BMI index was independently correlated with both prehypertension and hypertension [29]. These findings demonstrated that TyG-BMI index had great value in predicting cardiovascular events, but there still lacks the study on the association between TyG-BMI index and AF, especially in critically ill patients. Our study indicated that the TyG-BMI index could predict short- and long-term all-cause mortality in critically ill patients with AF, and L-shaped association between TyG-BMI index and all-cause mortality in critically ill patients with AF. Further, identifying high-risk populations in critically ill patients with AF might be useful in clinical trials. In subgroup analysis, critically ill patients with AF were shown to have lower all-cause mortality in the 30-day, 90-day, 180-day and 365-day periods when they had a higher level of TyG-BMI index, especially female, elderly patients (≥ 60 years) and the White patients. Meanwhile, higher levels of TyG-BMI had advantage for AF patients with or without hypertension and diabetes. Previous studies indicated that the IR could predicted the occurrence of AF in diabetic and non-diabetic population [30, 31]. One possible explanation was that the obesity status of patients with higher BMI weakened the harmful effect of IR represented by TyG on patients with AF, suggesting that the “obesity paradox” or “metabolically healthy obese” theory may exist in critically ill patients with AF, but there were few relevant studies on critically ill patients with AF at present. More clinical trials need be performed to investigate the link between TyG-BMI index and critically ill patients.

However, the theory of “metabolically healthy obesity (MHO)” is still controversial. HyunJung Lee et al. found that MHO patients were at increased risk for AF development, and obesity was independently associated with higher risk of AF [32]. But a study also reported that MHO and metabolically unhealthy obese (MUO) increased AF risk to a similar extent. Severity of obesity was positively associated with AF risk regardless of metabolic status [33]. These studies almost suggested that obesity was the independent risk factor of the occurrence and development of AF and the theory of MHO could not reasonably explain the conclusion of our study.

Interestingly, recent studies had also reported that the “obesity paradox” has been observed in the short- and long-term outcomes of critical illnesses [34]. Thus, the “obesity paradox” theory could be good and reasonable explanation for the opposite associations between TyG-BMI index and outcomes in our study. Although obesity have been proved to be an established risk factor for AF, there are conflicting opinions on the relationship between obesity and the prognosis of patients with AF [35]. Recent studies on AF revealed that weight-loss could reduce the occurrence and progression of AF and improve the prognosis of patients with AF. However, some studies indicated that the changes in body weight did not affect the prognosis of patients with AF. Interestingly, obesity was also significantly associated with favorable prognosis with AF. Liu et al. performed an exposure-effect meta-analysis and indicated that underweight was associated with a worse prognosis, but overweight and obesity were associated with improved outcomes in patients with AF [36].

The “obesity paradox” in AF could be elucidated with several potential mechanisms. Firstly, it has been reported in the literature that overweight and obese patients have higher blood pressure and heart rate, which allows them to tolerate faster, higher-dose therapies such as beta-blockers, ACEIs, and ARBs, all of which have the potential to improve the prognosis of patients with AF [37]. Secondly, atrial fibrosis and electrical remodeling in patients with AF are strongly associated with activation of the renin-angiotensin system. Renin-angiotensin levels may be elevated in overweight and obese patients compared with patients with normal BMI, which may also improve long-term cardiovascular prognosis [38]. Thirdly, atrial natriuretic peptide levels are significantly elevated in AF and could predict mortality of HF in patients with AF. Low circulating natriuretic peptide levels may also be associated with better prognosis in obese individuals [39, 40]. Finally, elevated levels of inflammatory markers can contribute to the onset and prognosis of AF. Among these markers, elevated circulating levels of tumor necrosis factor alpha (TNFα) can increase pulmonary venous arrhythmogenicity, leading to inflammation-associated AF. In the obese state, adipose tissue could produce TNFα receptors, which may maintain a favorable antiarrhythmic milieu in overweight or obese patients with AF [41, 42].

Our study indicates that TyG-BMI index, which combines TyG index and BMI, is an efficient clinical surrogate marker of critically ill patients with AF. The management of critically ill patients in ICU is an important topic and also the focus of clinical work. As an easy parameter to obtain when patients are admitted to ICU, TyG-BMI index can better prompt clinicians to identify high-risk patients in time, reduce mortality and improve patient’s prognosis.

There still existed several limitations in our study. Firstly, the retrospective analysis was a single-center study which was based on the observational data extracted from MIMIC-IV database, it is not easy to establish a determined causal relationship. Although a variety of variables were adjusted and subgroup analysis was conducted, we could not completely exclude the influence of potential confounders on the outcome. Secondly, the sample size of our study was moderate, and the results of cohort study with large sample size are needed to support our research conclusions. Thirdly, the data of blood glucose and lipids were extracted as the first measurements of patients who admitted to ICU, it was not completely certain that the measurements were obtained from fasting patients. Fourthly, our study did not demonstrate the biological plausibility of the relationship between the TyG-BMI index and all-cause mortality in critically ill patients with AF. Furthermore, we could not clarify when AF occurred and the main cause of death, these would reduce clinical relevance of the present analysis.

## Conclusion

The present study found that the TyG-BMI index was a potential predictor of 30-day, 90-day, 180-day and 365-day all-cause mortality in critically ill patients with AF. Moreover, L-shaped association existed between the level of TyG-BMI index and the risk of all-cause mortality in critically ill patients with AF. TyG-BMI index could be used as a valid indicator for grading and treating patients with AF in the ICU.

### Electronic supplementary material

Below is the link to the electronic supplementary material.

Supplementary Figure 1. Kaplan-Meier survival analysis curves for all-cause mortality. Kaplan-Meier curves and cumulative incidence of 90-day (A) and 180-day (B) all-cause mortality stratified by TyG-BMI index.

Supplementary Figure 2. Restricted cubic spline regression analysis of TyG-BMI index with all-cause mortality. Restricted cubic spline regression analysis of TyG-BMI index with 90-day (A) and 180-day (B) all-cause mortality.

Supplementary Figure 3. Forest plots of stratified analyses of TyG-BMI index and 90-day all-cause mortality.

Supplementary Figure 4. Forest plots of stratified analyses of TyG-BMI index and 180-day all-cause mortality.

Supplementary Material 5

Supplementary Material 6

## Data Availability

No datasets were generated or analysed during the current study.
