# Supplementary figures and images for: The association between triglyceride glucose-body mass index and all-cause mortality in critically ill patients with atrial fibrillation: a retrospective study from MIMIC-IV database
Source: Cardiovasc Diabetol. 2024 Feb 10;23:64. doi: 10.1186/s12933-024-02153-x (PMC10859027; doi:10.1186/s12933-024-02153-x)

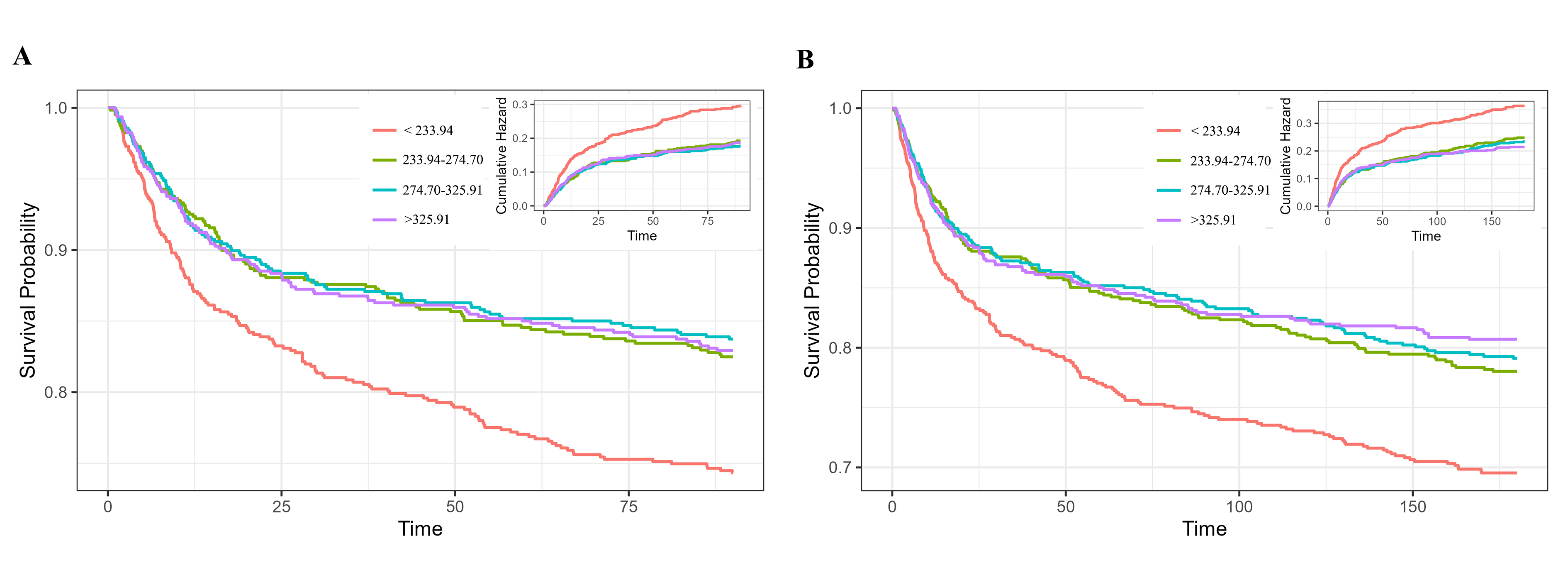

Supplement: Supplementary file 1 — Supplementary Figure 1. Kaplan-Meier survival analysis curves for all-cause mortality. Kaplan-Meier curves and cumulative incidence of 90-day (A) and 180-day (B) all-cause mortality stratified by TyG-BMI index. [file 12933_2024_2153_MOESM1_ESM.png]

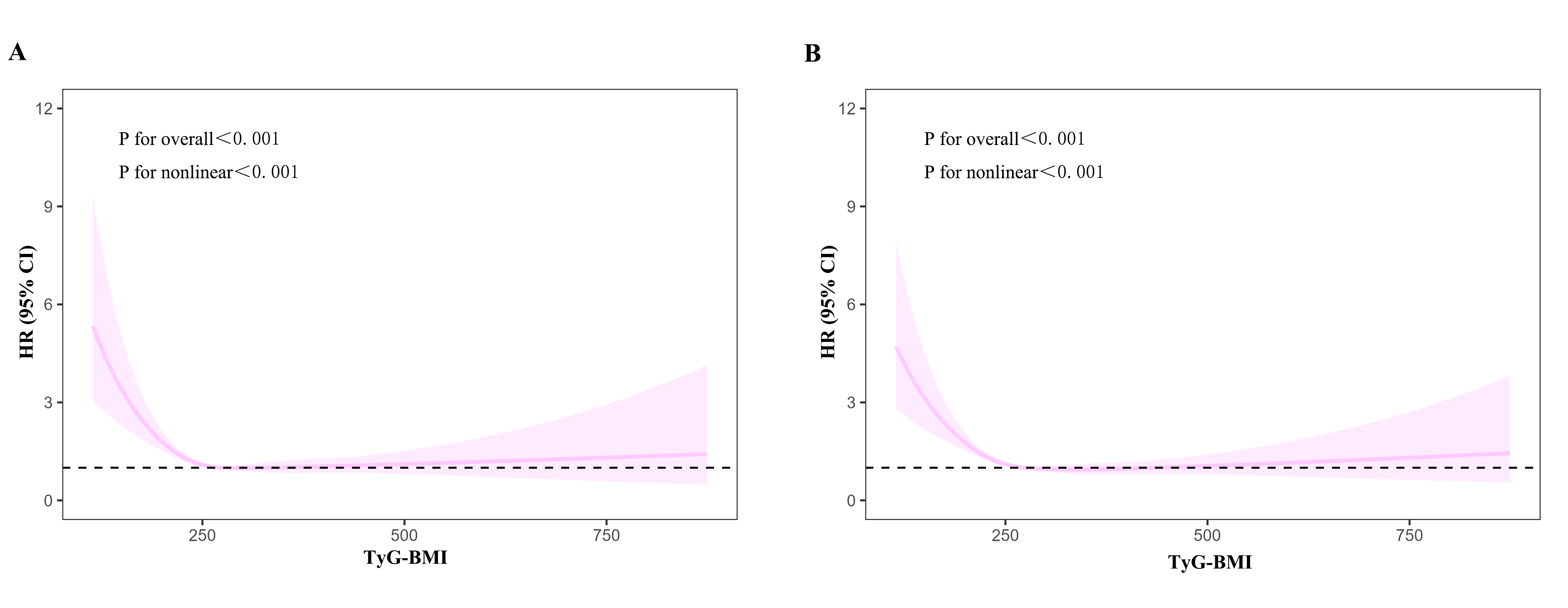

Supplement: Supplementary file 2 — Supplementary Figure 2. Restricted cubic spline regression analysis of TyG-BMI index with all-cause mortality. Restricted cubic spline regression analysis of TyG-BMI index with 90-day (A) and 180-day (B) all-cause mortality. [file 12933_2024_2153_MOESM2_ESM.png]

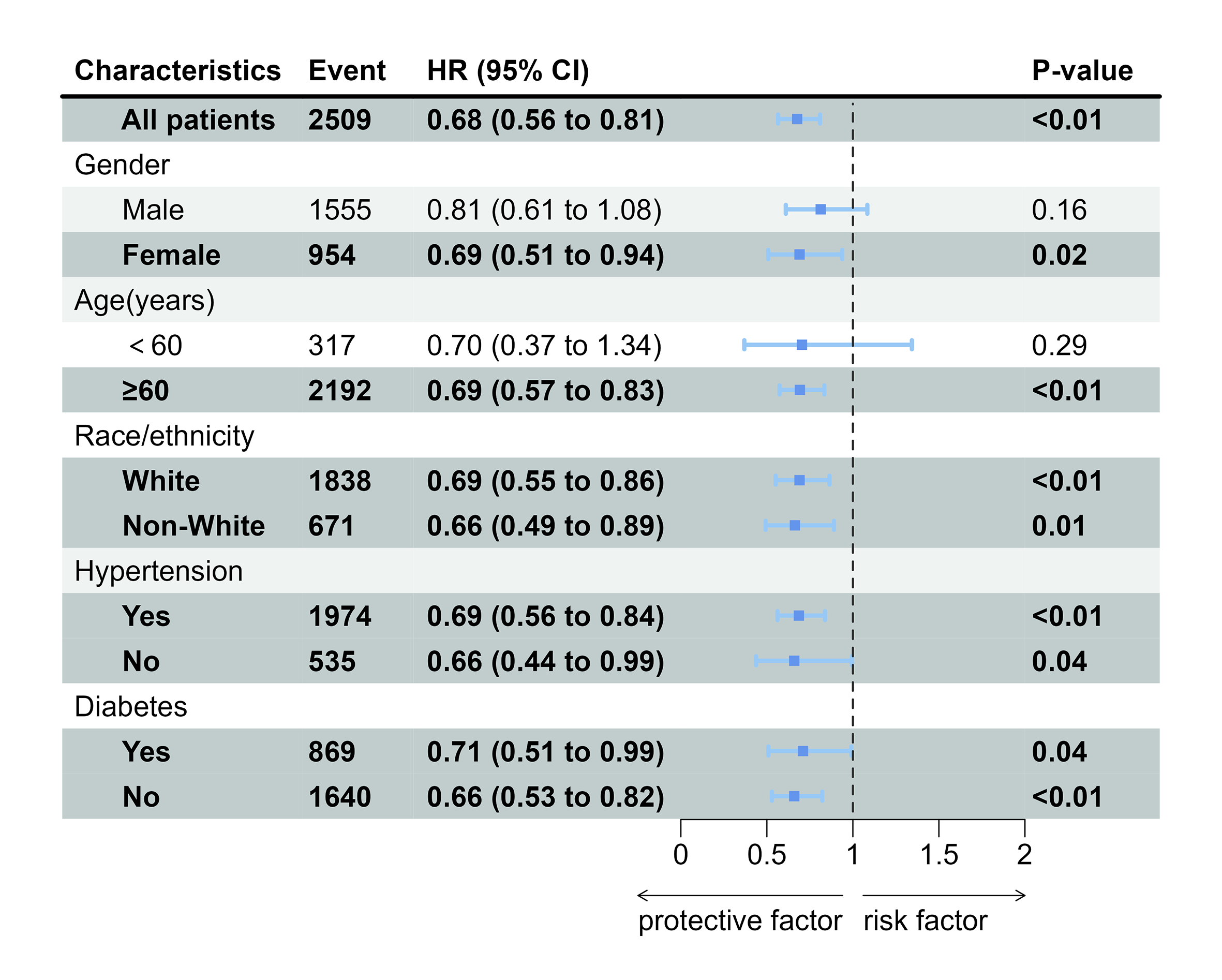

Supplement: Supplementary file 3 — Supplementary Figure 3. Forest plots of stratified analyses of TyG-BMI index and 90-day all-cause mortality. [file 12933_2024_2153_MOESM3_ESM.png]

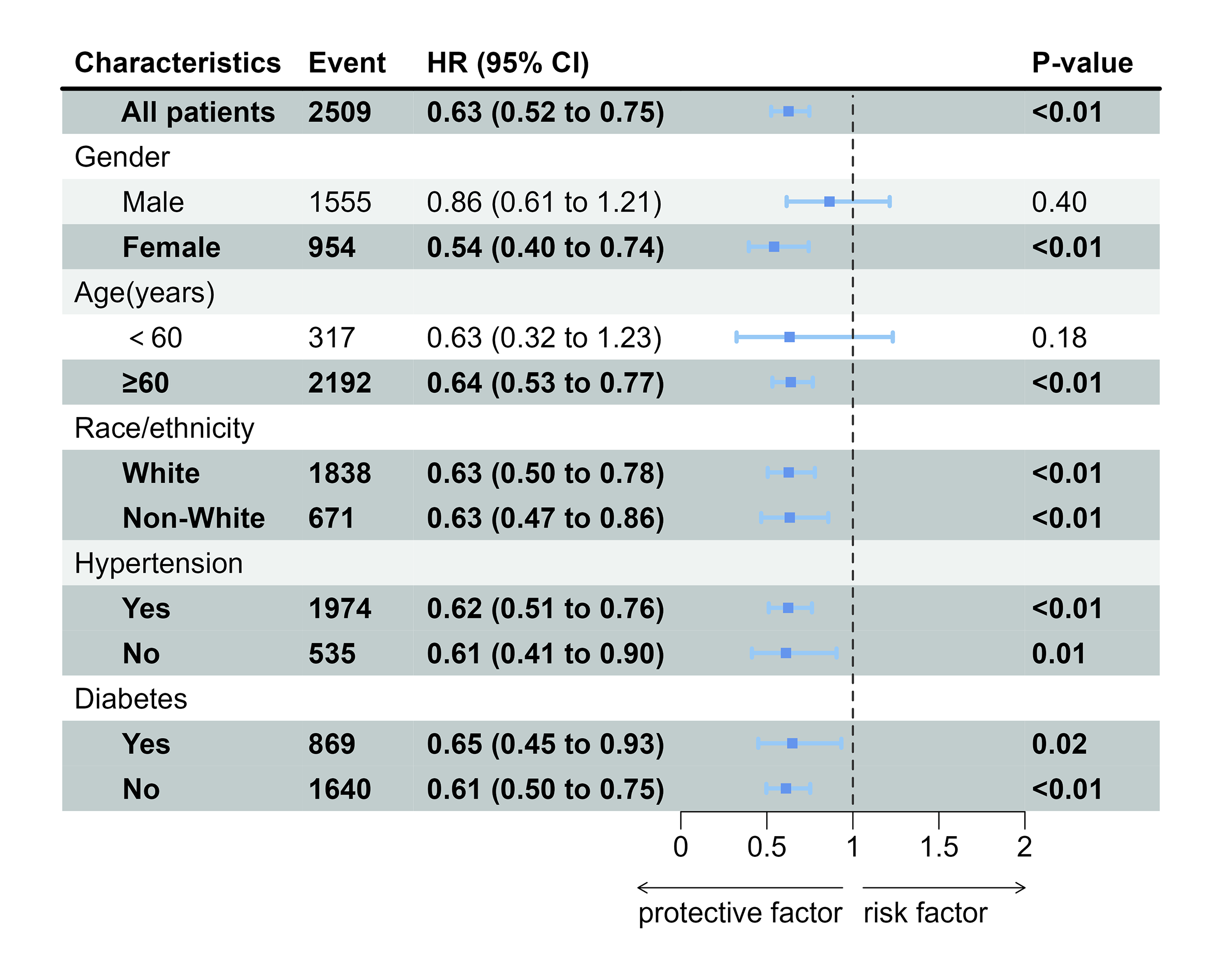

Supplement: Supplementary file 4 — Supplementary Figure 4. Forest plots of stratified analyses of TyG-BMI index and 180-day all-cause mortality. [file 12933_2024_2153_MOESM4_ESM.png]
