# Supplementary material for: The association between triglyceride glucose-body mass index and all-cause mortality in critically ill patients with atrial fibrillation: a retrospective study from MIMIC-IV database
Source: Cardiovasc Diabetol. 2024 Feb 10;23:64. doi: 10.1186/s12933-024-02153-x (PMC10859027; doi:10.1186/s12933-024-02153-x)
Supplement: Supplementary file 5 — Supplementary Material 5 [file 12933_2024_2153_MOESM5_ESM.docx]

**Supplementary Table 1 Cox proportional hazard models for 90-day and 180-day all-cause mortality.**

|  | **TyG-BMI index** | | | |
| --- | --- | --- | --- | --- |
|  | < 233.94 | 233.94-274.70 | 274.70-325.91 | >325.91 |
| **90-day mortality** |  |  |  |  |
| Number of deaths (%) | 161 (25.64) | 110 (17.52) | 102 (16.24) | 107 (17.04) |
| Model 1  HR (95% CI) P-value | 1.00 | 0.65 (0.51-0.83) <0.01 | 0.60 (0.47-0.77) <0.01 | 0.63 (0.50-0.81) <0.01 |
| Model 2  HR (95% CI) P-value | 1.00 | 0.71 (0.54-0.94)  0.02 | 0.66 (0.49-0.88) <0.01 | 0.67 (0.50-0.91)  0.01 |
| **180-day mortality** |  |  |  |  |
| Number of deaths (%) | 191 (30.46) | 138 (22.00) | 131 (20.89) | 121 (19.30) |
| Model 1  HR (95% CI) P-value | 1.00 | 0.68 (0.55-0.85) <0.01 | 0.64 (0.51-0.80) <0.01 | 0.60 (0.47-0.75) <0.01 |
| Model 2  HR (95% CI) P-value | 1.00 | 0.75 (0.58-0.96) 0.03 | 0.69 (0.53-0.90) <0.01 | 0.61 (0.46-0.81) <0.01 |

Model 1 Univariate model.

Model 2 adjusted for Age, Gender, Race, Heart rate, Hypertension, Diabetes, Insulin, Beta blockers, Statin, Amiodarone, Digitalis, pH, PT, Platelets, Chloride, Potassium, RBC, WBC, BUN, Creatinine, SOFA.
