# Supplementary material for: The association between triglyceride glucose-body mass index and all-cause mortality in critically ill patients with atrial fibrillation: a retrospective study from MIMIC-IV database
Source: Cardiovasc Diabetol. 2024 Feb 10;23:64. doi: 10.1186/s12933-024-02153-x (PMC10859027; doi:10.1186/s12933-024-02153-x)
Supplement: Supplementary file 6 — Supplementary Material 6 [file 12933_2024_2153_MOESM6_ESM.docx]

**Supplementary Table 2 Threshold effect analysis of TyG-BMI index on 90-day and 180-day all-cause mortality in AF patients.**

|  | **HR (95% CI), *P*-value** |
| --- | --- |
| **90-day mortality** |  |
| Fitting by the standard linear model | 0.998 (0.997-0.999) <0.01 |
| Fitting by the two-piecewise linear model |  |
| Infection point | 258.23 |
| TyG-BMI < 258.23 | 0.989 (0.986-0.993) <0.01 |
| TyG-BMI ≥ 258.23 | 1.001 (0.999-1.002) 0.46 |
| P for Log-likelihood ratio | <0.01 |
| **180-day mortality** |  |
| Fitting by the standard linear model | 0.998 (0.997-0.999) <0.01 |
| Fitting by the two-piecewise linear model |  |
| Infection point | 229.85 |
| TyG-BMI < 229.85 | 0.985 (0.981-0.990) <0.01 |
| TyG-BMI ≥ 229.85 | 1.000 (0.998-1.001) 0.48 |
| P for Log-likelihood ratio | <0.01 |
